# Nano-CaO_2_-Modified Biochar for Enhancing Thermophilic Anaerobic Digestion of Tofu Wastewater: A Review of Risk Mitigation and Resource Recovery Strategies

**DOI:** 10.3390/molecules31111882

**Published:** 2026-05-31

**Authors:** Xingzhong Zheng, Ndungutse Jean Maurice, Halima Niyilolawa Giwa, Abdulmoseen Segun Giwa

**Affiliations:** 1 School of Economics and Management, China University of Geosciences, Wuhan 430074, China; loulangushi@126.com; 2 College of Resources and Environment, Southwest University, Chongqing 400716, China; 3 Department of Biochemistry, College of Natural and Applied Sciences, Oduduwa University Ipetumodu, Ile Ife P.M.B. 5533, Nigeria; gsegon2@yahoo.com; 4 School of Civil and Environmental Engineering, Nanchang Institute of Science and Technology, Nanchang 330108, China

**Keywords:** tofu wastewater, nano-calcium-peroxide-modified biochar, thermophilic anaerobic digestion, risk management, failure mode and effects analysis, resource recovery, circular bioeconomy

## Abstract

Tofu wastewater (TWW), characterized as a high-strength organic effluent with elevated chemical oxygen demand (COD) and low pH, presents significant environmental challenges, including eutrophication, soil degradation, and greenhouse gas emissions. Conventional disposal methods have proven inadequate in mitigating these risks; however, thermophilic anaerobic digestion (TAD) has emerged as a viable technology for bioenergy recovery. Nonetheless, TAD is impeded by rapid acidification, ammonia and hydrogen sulfide inhibition, and the accumulation of volatile fatty acids (VFAs). This review introduces nano-calcium-peroxide-modified biochar (nano-CaO_2_/BC) as a multifunctional additive designed to establish an integrated framework for intervention, risk mitigation, and resource recovery. The proposed amendment synergistically combines the adsorptive and microbial-supportive properties of biochar with the controlled oxidative and alkaline characteristics of nano-CaO_2_. Under thermophilic conditions, the slow hydrolysis of nano-CaO_2_ generates transient microaerobic zones that enhance polymer hydrolysis, suppress ammonia (NH_3_) and hydrogen sulfide (H_2_S) formation, and facilitate the oxidation of inhibitory VFAs, concurrently releasing calcium hydroxide (Ca(OH)_2_) for sustained pH buffering. Utilizing failure mode and effects analysis (FMEA) as a semi-quantitative assessment tool, the results indicate that the composite significantly reduces risk priority numbers associated with acidification, ammonia toxicity, and sulfide inhibition when compared with conventional TAD methods. The resultant digestates, which are enriched in nutrients and recalcitrant carbon, possess the potential to serve as valuable soil amendments, thereby contributing to a circular bioeconomy. A techno-economic assessment grounded in unit cost analysis suggests that positive net benefits may be realized through enhanced biogas recovery and the mitigation of environmental penalties. However, empirical validation at the pilot scale is essential to substantiate the projected performance. This review underscores critical knowledge gaps and proposes a systematic experimental framework aimed at translating the conceptual risk mitigation strategy into practical applications.

## 1. Introduction

The global tofu industry, particularly in East and Southeast Asia, generates substantial environmental burdens through tofu wastewater (TWW). TWW is characterized by elevated biochemical oxygen demand (BOD) and chemical oxygen demand (COD), acidic pH, high suspended solids, significant nitrogen and phosphorus loads, and soluble carbohydrates leached during coagulation [1,2,3,4]. From a risk-governance perspective, TWW embodies multiple overlapping hazards: pollution-diffusion risks (water and soil contamination from high COD and acidity), process-failure risks (biological treatment collapse due to acidification, sNH_3_, and H_2_S inhibition), and resource-waste risks (foregone energy recovery from labile organics) [5]. Market projections indicate that the global tofu industry will grow from USD 3.94 billion in 2025 to USD 7.07 billion in 2032, with China dominating both consumption and production [6]. Wastewater generation approximates 7–10 L per kg of tofu produced; Indonesian data show that 84,000 factories processing 2.56 million tons of soybeans annually collectively generate approximately 2.0 × 10^7^ m^3^ of TWW [3]. Extrapolating to China’s 972 tofu enterprises yields an estimated annual TWW generation of 1.944 × 10^10^ m^3^. According to Feng et al. [1] conventional disposal methods such as landfilling, composting, and direct discharge remain fundamentally inadequate, causing rapid dissolved oxygen depletion, eutrophication, soil acidification, and uncontrolled greenhouse gas emissions—all unmanaged pollution-diffusion pathways [7].

Thermophilic anaerobic digestion (TAD, 50–60 °C) offers enhanced kinetics, pathogen destruction, and volumetric biogas productivity [8]. However, TAD of TWW exhibits heightened sensitivity to environmental perturbations; accelerated acidogenesis combined with high organic loads induces rapid VFA accumulation and pH depression, thereby severely inhibiting methanogenic archaea [9]. This constitutes a high-probability, high-impact process-failure risk. It must be emphasized that no direct experimental studies on TAD of TWW currently exist in the literature; therefore, performance insights are extrapolated from analogous high-strength substrates (e.g., blackwater, food waste, sewage sludge). Elevated nitrogen content further generates free ammonia nitrogen (NH_3_-N) at concentrations toxic to methanogens, compounding instability [4,10]. Quantitative evidence from comparative anaerobic digestion (AD) studies shows that tofu liquid waste yields 93.00 mL biogas/g COD versus 143.99 mL/g COD for tapioca-flour liquid waste, a 35.41% reduction attributable to TWW’s inherent inhibitory characteristics [11]. Process intensification through catalytic additives represents a strategic approach to overcoming thermodynamic and kinetic limitations. Biochar, produced via biomass pyrolysis, enhances AD through a high specific surface area, porosity, and adsorption capacity [12]. However, pristine biochar exhibits limited efficacy under thermophilic conditions, where enhanced mass transfer and intensified inhibition necessitate robust intervention. Nano-CaO_2_/BC substantially improves physicochemical properties, yielding increased surface area, porosity, alkaline functional groups, and buffering capacity that are advantageous under elevated-temperature operation [13,14].

Nano-CaO_2_/BC enables an integrated management framework of technical intervention, risk control, and resource recycling through multiple mechanisms: (i) controlled hydrolysis generating Ca(OH)_2_ and H_2_O_2_, with subsequent H_2_O_2_ decomposition providing sustained micro-aeration that maintains thermophilic kinetics while oxidizing recalcitrant compounds [15,16]; (ii) robust pH buffering from Ca(OH)_2_ counteracting accelerated acidogenesis [17]; (iii) adsorption of inhibitory NH_3_-N, H_2_S, and VFAs within the porous carbon matrix, reducing bioavailable concentrations below toxicity thresholds [18,19]; and (iv) biofilm-carrier functionality supporting syntrophic bacteria and methanogenic consortia adapted to elevated temperatures [20]. These synergistic mechanisms collectively address identified risk dimensions, achieving enhanced process stability, organic removal efficiency, and biogas yield while establishing a circular bioeconomy model wherein waste is valorized to renewable energy and nutrient-rich agricultural digestate [21,22]. This review systematically examines nano-CaO_2_/BC synthesis, physicochemical characterization, and its multifunctional role in revolutionizing TWW management through risk control and resource recovery optimization.

### Literature Search Approach

Data were collected from the relevant literature using major scientific databases, including Scopus, Web of Science, and Google Scholar. The keywords included combinations of “tofu wastewater,” “anaerobic digestion,” “biochar,” “calcium peroxide,” “nanomaterials in wastewater treatment,” and “risk assessment in anaerobic digestion,” using Boolean operators (AND, OR). Articles were screened for relevance regarding anaerobic digestion performance, inhibition control, risk mitigation strategies, and resource recovery mechanisms. The search prioritized recent peer-reviewed articles that are pertinent to the multidisciplinary scope of the work. This review does not claim to be a PRISMA-compliant systematic review; rather, it is a targeted, hypothesis-driven critical review that synthesizes fragmented literature to propose a novel risk-management framework for an understudied wastewater stream. The non-systematic approach aligns with the PRISMA 2020 statement [23], which provides guidelines for systematic reviews but is not applicable here given the exploratory and hypothesis-generating nature of the work.

## 2. Interdisciplinary Theories of Risk Management and Environmental Engineering

### 2.1. Core Theory Integration for TWW Treatment

The application of nano-CaO_2_/BC for TAD of TWW requires an interdisciplinary framework integrating risk-management science with environmental engineering principles. Three complementary theories establish the foundation: Risk Matrix Theory for hazard prioritization, whole-process Risk Management Theory for systematic control, and Risk–Benefit Synergy Theory under circular economy principles for value optimization [24,25,26]. In this review, risk is formally defined as the product of the probability of an undesirable event and the magnitude of its consequences on environmental, operational, and economic outcomes. Risk assessment integrates four components: (i) hazard identification through physicochemical characterization of TWW, (ii) probability assessment using kinetic and thermodynamic analysis, (iii) consequence assessment via performance metrics such as methane yield reduction and environmental impact, and (iv) risk characterization through semi-quantitative failure mode and effects analysis (FMEA). Risk Matrix Theory classifies TWW treatment hazards along probability and impact dimensions [24]. Under thermophilic conditions, process failure via acidification constitutes a high-probability, high-impact risk: accelerated hydrolysis of TWW’s soluble carbohydrates can overwhelm methanogenic capacity within hours, causing VFA accumulation, catastrophic pH depression, and irreversible archaeal inhibition [5,11]. Equipment corrosion from biogenic H_2_S represents a medium-probability, high-impact risk; thermophilic operation increases sulfate-reduction rates and H_2_S volatility, potentially reducing metallic infrastructure lifespan by 30–50% [19]. Ammonia inhibition represents a medium-probability, medium-impact risk: TWW total nitrogen combined with elevated temperature shifts the NH_3_/NH_4_^+^ equilibrium toward toxic unionized ammonia, reducing methane yields without complete process failure [10]. Pathogen survival in digestate constitutes a low-probability, medium-impact risk; thermophilic retention >10 days typically achieves Class A biosolids standards, though temperature fluctuations warrant monitoring [8].

Whole-process Risk Management Theory segments TWW treatment into discrete control nodes with corresponding objectives [25]. The generation node addresses wastewater variability [2,4], requiring characterization and homogenization. The preprocessing node targets feed conditioning (solids management, pH adjustment, and temperature equilibration) to prevent cold shock and hydrodynamic perturbations. The TAD reaction node represents the critical control point: maintaining pH 7.2–8.0, VFA < 2000 mg/L (acetic acid equivalents), free NH_3_ < 100 mg/L, and H_2_S mitigation to prevent microbial inhibition and infrastructure corrosion [8]. The digestate utilization node balances pathogen control, nutrient runoff prevention, and soil improvement value [27]. Risk–Benefit Synergy Theory under circular economy principles posits synchronous optimization of risk control and resource recovery, transforming mitigation expenditures into value-generating investments [26]. Enhanced carbonaceous COD removal and effluent stabilization directly correlate with biogas generation (0.98 L CH_4_/g C removed), where risk reduction yields energy revenue [5]. Process stabilization via pH buffering and inhibitor removal maximizes productivity, while nutrient-rich digestate suitable for agricultural application replaces synthetic fertilizers and improves soil health [19,28]. This coupling fundamentally repositions TWW treatment from a cost-center to a profit-generating activity.

### 2.2. Risk Identification Framework for TWW Treatment

A systematic risk-identification framework for TAD of TWW must catalog three overlapping hazard categories: pollution-diffusion risks, process-failure risks, and resource-waste risks. This framework establishes the foundation for targeted intervention design. Pollution-diffusion risks encompass environmental receptor damage from uncontrolled TWW discharge. Water-pollution risk scales directly with COD concentration; TWW exhibiting 4017–8500 mg COD/L [2,4], represents an oxygen demand equivalent to 3–4 kg O_2_/m^3^, sufficient to deoxygenate 300–400 m^3^ of receiving water per m^3^ discharged under typical stream reaeration. Nutrient loads (total N 591.8 ± 238.2 mg/L; P 2.56–95.50 mg/L) trigger eutrophication and cyanotoxin production [1,4,7]. Soil-pollution risk manifests through progressive acidification from raw TWW pH (4.82–5.50) [4] and mobilizes phytotoxic Al and Mn while reducing P and base cation availability [7]. Sodium accumulation degrades soil structure, reducing infiltration and promoting surface crusting [29]. Excessive organic loading induces anaerobic soil conditions, promoting denitrification and greenhouse gas emissions. Process-failure risks represent operational hazards specific to TAD of TWW. Rapid acidification risk arises from kinetic mismatch between acidogenic and methanogenic consortia under thermophilic conditions [5]. Soluble carbohydrates (stachyose, raffinose, sucrose) undergo fermentation by acidogens with generation times of hours, producing VFAs that accumulate faster than methanogenic archaea (generation times of days) can consume them [11]. This creates a positive feedback loop: VFA accumulation depresses pH, inhibiting methanogens more severely than acidogens [30]. Thermophilic operation intensifies this risk because hydrolysis and acidification rates increase more sharply with temperature than methanogenic rates, widening the kinetic gap [31].

The NH_3_ inhibition risk derives from protein deamination. At thermophilic temperatures, the NH_3_/NH_4_^+^ equilibrium shifts toward unionized NH_3_, which diffuses across cell membranes, disrupting proton gradients and intracellular pH [10]. Acetoclastic methanogens exhibit inhibition thresholds of 50–100 mg NH_3_-N/L under thermophilic conditions versus 150–300 mg/L mesophilically [32]. Hydrogen sulfide toxicity risk manifests through sulfate oxidases, denatures proteins, and partitions into the gas phase, corroding metallic infrastructure while reducing biogas CH _4_ content [19]. Thermophilic conditions increase both sulfate-reduction rates and H_2_S toxicity. Resource-waste risks represent foregone circular-economy opportunities. Energy-recovery waste: TWW COD represents a potential methane production of 0.35–0.50 m^3^ CH_4_/kg COD degraded [11,33]. For a typical facility processing 30 metric tons soybeans annually generating 2.0 × 10^7^ m^3^ TWW, foregone energy equals approximately 7.0 × 10^6^ m^3^ CH_4_ annually, sufficient for 10,000–15,000 households [5]. Nutrient-recovery waste: total N (591.8 mg/L) and P (2.56–95.50 mg/L) [1,4] discharge represents phosphorus losses of 0.5–2.0 kg P per ton TWW, which is significant given global phosphate-rock depletion [28]. Water-recovery waste accompanies these losses, as treatable volumes suitable for reuse are discharged without recovery in water-stressed regions where many facilities operate [3,6]. This framework establishes TAD of TWW as a multi-risk management problem requiring integrated solutions.

## 3. Thermophilic Anaerobic Digestion of Tofu Wastewater: Process Fundamentals and Risk Characterization

### 3.1. Physicochemical Properties of Tofu Wastewater as Risk Determinants

TWW exhibits a variable yet consistently high organic load, dictating both energy-recovery potential and process-failure risks in thermophilic TAD [2,4,10]. Its composition—predominantly soluble carbohydrates (stachyose, raffinose, sucrose), proteins, and minor lipids, coupled with a BOD:COD ratio >0.5—confirms exceptional biodegradability but inherent acidification susceptibility [2,5,11]. Thermophilic anaerobic digestion conditions accelerate the hydrolysis of readily biodegradable substrates, driving rapid VFA production [31]. This intensifies a critical kinetic imbalance between fast acidogenesis and slow methanogenesis in which VFA accumulation exceeds consumption, depressing pH, and inhibiting acetoclastic archaea [5]. Sintawardani et al. [9] noted that soluble fermentable oligosaccharides are the primary acidification drivers, rendering TWW mono-digestion intrinsically high-risk without engineered pH buffering and microbial stabilization. Table 1 presents physicochemical characteristics of TWW from different soybeans.

The compositional variability of TWW, quantified across soybean varieties by Hardyanti et al. [2], establishes feedstock-driven parameters that critically influence process stability in TAD. TSS ranges from 218 to 444 mg/L, a twofold variation affecting solids loading and reactor hydrodynamics. COD ranges from 4017 to 4583 mg/L (14% variation), cumulatively impacting organic loading rate stability in continuous systems [5]. BOD exhibited a more pronounced range of 2255–3481 mg/L (54% variation), reflecting differences in the biodegradable fraction that directly govern methane potential [11]. NH_3_-N demonstrates the most substantial variability: 8.61–13.86 mg/L (61% range). Notably, samples from Wonogiri soybeans show elevated nitrogen content, increasing ammonia inhibition risk under TAD via the temperature-dependent NH_3_/NH_4_^+^ equilibrium shift toward toxic unionized ammonia [10,17]. The Wonogiri variety simultaneously exhibited maximal TSS, COD, BOD, and NH_3_-N concentrations [2], confirming soybean selection as a primary determinant of wastewater risk profiles. This aligns with Feng et al. [1], who characterized concentrated TWW at a mean COD of 21,894 ± 11,485 mg/L, total nitrogen of 591.8 ± 238.2 mg/L, and a C/N ratio of 36.9 ± 7.4. While this elevated C/N ratio, combined with readily biodegradable sugars and proteins, provides a favorable methanogenic substrate, it simultaneously creates kinetic imbalance risks as previously identified [34]. Such feedstock-driven variability establishes a risk-management imperative: systems designed for mean feed characteristics must incorporate robust buffering capacity and inhibitor management to accommodate compositional extremes and prevent cumulative destabilization. Different properties of TWW from tofu processing facilities are shown in Table 2.

Table 2 presents data from Faisal et al. [4], quantifying facilities-level TWW variability, revealing processing-driven compositional extremes that amplify TAD risks. pH ranges from 4.82 to 5.50, consistently acidic, favoring acidogenic bacteria over methanogens from the outset and necessitating active buffering intervention [4,9]. MLSS varied dramatically (1050–3130 mg/L; 198% range), reflecting differential solids retention during processing. COD exhibited 5000–8500 mg/L (70% variation), directly impacting organic loading rate calculations and biogas potential [5]. NH_3_-N demonstrates extreme variation: 33.50–129.30 mg/L (286% range), with the Tahu Sumedang facility concentrations approaching inhibitory thresholds prior to thermophilic concentration effects [10]. Phosphate (PO_4_-P) varies from 0.97 to 95.50 mg/L (98-fold range), reflecting coagulant use and phosphorus management practices that affect both nutrient recovery potential and struvite precipitation risk in reactor infrastructure [28]. Turbidity ranged between 387 and 921 NTU, indicating substantial suspended solids content affecting reactor hydrodynamics and mass transfer [4]. Thus, TWW is thermodynamically predisposed toward TAD, while simultaneously presenting intensified process risks that require robust intervention strategies.

### 3.2. Thermophilic Anaerobic Digestion Process: Mechanisms and Risk Amplification

**Figure 1:**
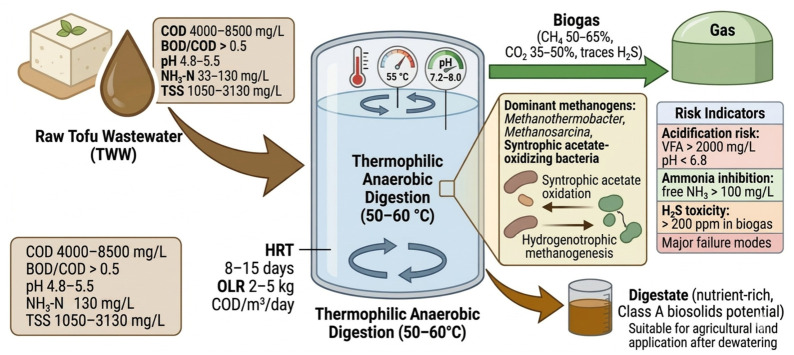
Thermophilic anaerobic digestion of Tofu wastewater.

TAD is thermodynamically aligned with TWW treatment, transforming high-strength organic pollutants into biogas and nutrient-rich digestate [8,31]. Moerland et al. [27] reported that TAD offers higher maximum specific growth rates for hydrolytic and acidogenic bacteria than for hyper-thermophilic or mesophilic conditions, accelerating hydrolysis of particulate organics and enabling shorter hydraulic retention times with reduced reactor volumes [8]. Superior pathogen destruction produces digestate meeting high biosolids standards, expanding agricultural utilization pathways [35]. Given the absence of direct studies on the TAD of TWW, this analysis extrapolates treatment efficacy by examining TAD performance on substrates with analogous physicochemical characteristics: high-strength blackwater (COD > 15,000 mg/L, high protein content, C/N ratio 30–40) and sewage sludge (high organic solids, complex microbial communities) [27,36]. Although these substrates differ from TWW in certain details, their similar challenges with ammonia inhibition, VFA accumulation, and the need for efficient syntrophic metabolism make them the most relevant available comparators. Zhang et al. [36] demonstrated that in a thermophilic upflow anaerobic sludge blanket reactor treating blackwater, the cessation of effluent recirculation significantly enhanced performance. Without recirculation, methanogenesis efficiency increased from 45.0 ± 2.9% to 56.7 ± 5.5%, while COD accumulation within the reactor dropped sharply from 17.2% to 3.8%. This improvement was linked to reduced VFA in the effluent (from 0.64 ± 0.18 to 0.15 ± 0.10 g/L) and a pronounced microbial community shift, in which *Methanosarcina* became dominant over *Methanothermobacter*. This transition facilitated syntrophic acetate oxidation and hydrogenotrophic methanogenesis, suggesting that minimizing hydraulic mixing fosters closer syntrophic cooperation essential for processing protein- and lipid-rich waste [36]. Figure 1 shows TAD of TWW.

Further supporting the applicability of TAD to high-strength waste, Moerland et al. [27] achieved 70% COD removal and 62% methanization during TAD of concentrated blackwater at a high organic loading rate (OLR) of >3 kg COD/m^3^/day with an 8.7-day retention time. Notably, the process exhibited a rapid 12-day start-up and a metabolic shift toward syntrophic acetate oxidation, a pathway often prevalent under TAD when acetoclastic methanogenesis is inhibited by ammonia or VFA, which are common in proteinaceous waste like tofu effluent [11]. In addition to organic conversion, TAD offers a critical advantage in pathogen inactivation, which is a key consideration for safe resource recovery. Al-Sulaimi et al. [37] reported a 51.43% inactivation efficiency of viable helminth ova (predominantly *Ascaris*) in sewage sludge over 60 days of TAD, alongside 34% COD removal. Seruga et al. [35] further demonstrated the rapid elimination of pathogens during TAD, with *Salmonella* Senftenberg W775 inactivated within 6.06 h and *Ascaris suum* eggs within approximately 10 h. Their full-scale validation in a Kompogas^®^ reactor confirmed process stability, maintaining a pH of 8.5 and total solids of 35%, which contributed to the consistent hygienization of the digestate. Collectively, these findings indicate that TAD not only achieves robust COD reduction and methane production from high-solid wastes but also ensures substantial pathogen destruction. This dual functionality makes TAD a technically viable and promising strategy for treating challenging agro-industrial effluents like TWW, meriting direct investigation.

In contrast, the TAD of TWW is intensified by the decomposition of acetoclastic methanogens at thresholds of 50–100 mg NH_3_ [10,17]. Rapid acidogenesis of soluble sugars under TAD accelerates VFA overproduction, thereby widening the kinetic imbalance between acidogenesis and methanogenesis [5,9]. VFA accumulation depresses pH below methanogenic optima, creating positive feedback inhibition [36,38]. Sulfate-reducing bacteria outcompete methanogens for electrons, generating H_2_S that inhibits cytochrome oxidases, denatures proteins, corrodes infrastructure, and reduces biogas methane content [19,39]. Raw TWW acidity (pH 4.82–5.50) favors acidogens over methanogens from the start of the process [4]. Syaichurrozi et al. [11] quantified the process-failure risk via comparative anaerobic digestion: optimal co-digestion of tofu liquid waste (TLW) and tapioca-flour liquid waste (50:50 *v*/*v*, pH 7) yielded 341.13 mL biogas/g COD added, 48.1% methane, and 55% COD removal. TLW mono-digestion produced only 93.00 mL/g COD, a 35.41% reduction versus tapioca-flour liquid waste mono-digestion (143.99 mL/g COD), attributed to rapid acidification and NH_3_ accumulation inherent to TWW composition [11]. Under thermophilic conditions, differential temperature sensitivities and kinetic parameters create rate imbalances: accelerated acidogenesis of soluble carbohydrates overwhelms slower acetogenesis and methanogenesis, thereby inducing VFA accumulation, pH depression, and methanogenic inhibition. This high-probability, high-impact acidification failure mode characterizes TAD of TWW.

## 4. Assessment of Biochar as a Risk-Control Substrate

### 4.1. Physicochemical Properties of Biochar: Foundations for Risk Mitigation

**Figure 2:**
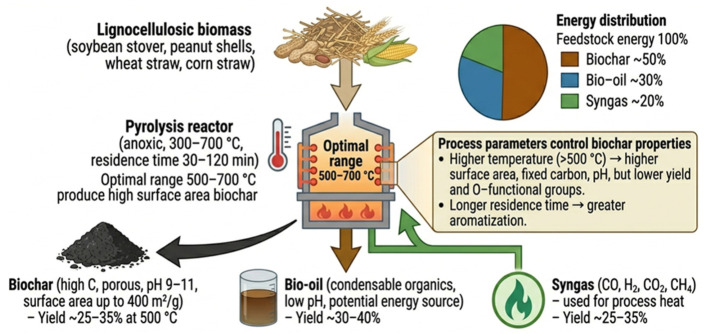
Production of biochar via pyrolysis of biomass.

Biochar is a porous, carbon-rich solid produced through pyrolysis, which is the thermal decomposition of organic and inorganic materials in the absence of oxygen at temperatures between 300 and 700 °C [18,40]. This process differs from torrefaction, which occurs at lower temperatures (200–300 °C) and mainly improves fuel properties [41]. Pyrolysis drives the depolymerization, dehydration, and aromatization of biopolymers, yielding solid biochar, condensable bio-oil, and non-condensable syngas [42]. Biochar’s physicochemical properties are governed by pyrolysis parameters, enabling optimization for specific TAD risk-control applications [18]. Feedstock selection is foundational: lignocellulosic biomass yields high-porosity structures supporting microbial colonization and adsorption, while manure-based feedstocks produce ash-rich biochars with elevated pH and nutrient content advantageous for acid buffering [43]. In addition, the pyrolysis temperature exerts dominant control over material properties. Higher temperatures (>500 °C) drive heteroatom volatilization, enriching fixed carbon and developing condensed polycyclic aromatic structures with enhanced biological stability and persistence in anaerobic digesters [40]. Thermal restructuring creates extensive microporous networks, increasing the specific surface area, which often exceeds 400 m^2^/g, thereby enhancing the adsorption capacity for inhibitory compounds and mitigating toxicity risks [18]. Lower temperatures (<400 °C) preserve aliphatic compounds and oxygen-containing functional groups, yielding higher cation exchange capacity (CEC) but reduced structural stability [40]. For TAD applications, the trade-off between surface functionality (optimal at intermediate temperatures) and structural stability (optimal at high temperatures) requires calibration to dominant risk profiles. Residence time governs carbonization completeness; extended durations facilitate aromatic cluster growth and pore-structure development, optimizing surface area and pore volume [42]. Gai et al. [18], identified 500 °C as optimal for maximizing CEC across tested biochars, with values declining at higher temperatures. Feedstock, peak temperature, and residence time thus synergistically tailor biochar surface functionality, CEC, pH, and adsorption affinity for process-failure risks in TAD. The application of biochar in AD has been comprehensively reviewed, highlighting its role in DIET, adsorption, and microbial support [44]. The production of biochar via the pyrolysis of biomass is illustrated in Figure 2.

Table 3 presents the physicochemical properties of biochar from different feedstocks and pyrolysis temperatures, revealing systematic relationships critical for TAD applications. At 700 °C, peanut-shell biochar achieves the highest surface area (448.20 m^2^/g) and pore volume (0.20 cm^3^/g), while soybean-stover biochar at the same temperature achieves 420.30 m^2^/g with a slightly lower pore volume (0.19 cm^3^/g). These high-surface-area biochars produced at elevated temperatures demonstrate pH values exceeding 10.5, providing substantial alkaline buffering capacity [40]. In contrast, biochars produced at 300–400 °C exhibited surface areas of only 3–10 m^2^/g but retained higher oxygen content (21–26%) and CEC, which is advantageous for nutrient retention. The elemental composition trends revealed decreasing O/C and H/C ratios with increasing pyrolysis temperature, indicating progressive aromatization and the formation of condensed carbon structures resistant to microbial degradation in the digester environment [18]. For thermophilic TAD applications, biochars produced at 600–700 °C from lignocellulosic feedstocks (peanut shells, soybean stover) appeared most suitable because of their combination of high surface area for inhibitor adsorption, elevated pH for acid buffering, and structural stability under elevated temperatures and mechanical stress conditions [40,42]. Wheat-straw biochar at 700 °C (107 m^2^/g, pH 9.20) and corn-straw biochar at 600 °C (7 m^2^/g, pH 10.40) demonstrate that feedstock selection significantly influences the trade-off between surface-area development and alkaline functional group retention [18]. Table 3 shows the physicochemical characteristics of biochar from different feedstocks.

Furthermore, strategic modification of biochar is critical for addressing the multifactorial risk profile of TAD of TWW. Engineered biochars modified through chemical, physical, or biological methods enhance inherent properties and introduce targeted functionalities that are absent in pristine materials [45,46,47]. For TAD risk control, modifications that enhance ammonia adsorption, provide alkaline buffering, or introduce oxidative capacity are particularly relevant. Li et al. [45] reported that magnesium–iron co-modified biochar (Mg/Fe-BC) achieved an NH_3_-N adsorption capacity of 87.83 mg/g, substantially exceeding Mg-BC (26.77 mg/g) and Fe-BC (14.57 mg/g). Addition of 5% Mg/Fe-BC to biogas slurry removed 38.57% of NH_4_^+^-N, directly mitigating ammonia inhibition risk. In chicken-manure anaerobic digestion, 2–5% Mg/Fe-BC doses increased cumulative methane production by 116–229%, stabilizing system parameters and enriching key methanogens [45]. Chiang et al. [46] reported calcium-oxide-modified biochar-enhanced alkalinity, nutrient retention, and adsorption capacity in vacuum-blackwater anaerobic digestion, fostering favorable microbial environments and improving biogas production while addressing multiple process-failure risks simultaneously. Zhang et al. [47] found that Fe_3_O_4_-BC (3.0 g/L) in erythromycin-wastewater anaerobic digestion achieved a maximum methane yield of 327.7 mL/g COD—a 55.7% increase versus controls. Strategic modification thus enables targeted risk control for thermophilic TWW treatment, where intensified acidification, ammonia inhibition, and sulfide generation demand multifunctional intervention. 

### 4.2. Modification of Biochar with Calcium Peroxide: Engineering for Multi-Risk Control

Nano-CaO_2_/BC overcomes the inherent limitations of pristine biochar for TAD of TWW, where finite adsorption capacity and lack of targeted reactivity for acidification, sulfide toxicity, and ammonia inhibition constrain performance [13,19]. Nano-CaO_2_/BC synthesis introduces a dual mechanism, including sustained chemical oxidation and pH modulation, that directly targets the amplified risks of thermophilic operation [14]. Upon introduction to aqueous TAD environments, nano-CaO_2_ undergoes controlled hydrolysis. The generated H_2_O_2_ decomposes to reactive oxygen species (ROS) via Fenton-like reactions, creating transient micro-aerobic zones within the bulk anaerobic matrix [19]. These ROS oxidize inhibitory compounds, providing alternative consumption pathways that supplement methanogenic activity and prevent accumulation, particularly under thermophilic conditions, where accelerated acidogenesis rapidly overwhelms methanogenic capacity [14]. Calcium peroxide has been extensively studied as a pretreatment agent to improve the AD of waste [48].

**Figure 3:**
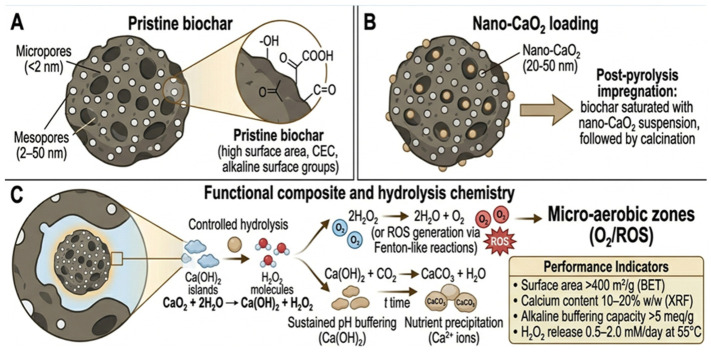
The modification of biochar with nano-calcium peroxide: (**A**) pristine biochar with functional groups, (**B**) nano-CaO_2_, deposition via post-pyrolysis impregnation, (**C**) hydrolysis and carbonation reactions that generate alkaline buffering, reactive oxygen species, and calcium-rich mineral phases for comprehensive risk control in thermophilic anaerobic digestion.

Concurrently, hydrolysis-derived Ca(OH)_2_ elevates localized pH, counteracting acidification from VFA accumulation and shifting the NH_3_/NH_4_^+^ equilibrium toward less toxic NH_4_^+^ via Le Chatelier’s principle [17,49]. This buffering effect is essential under thermophilic conditions where temperature-dependent equilibrium shifts would otherwise increase toxic unionized ammonia fractions. Liberated calcium ions further immobilize phosphorus and nitrogen through precipitation as hydroxyapatite and ammonium calcium phosphate minerals, sequestering nutrients in digestate for agricultural valorization while removing them from the liquid phase where they contribute to inhibition or eutrophication risk [21,22,28]. This synergistic action transforms biochar from a passive adsorbent to an active system-stabilizing catalyst for advanced TAD, systematically addressing the multiple risk dimensions of TWW treatment via TAD. Figure 3 shows the modification of biochar with nano-CaO_2_.

Nano-CaO_2_/BC synthesis proceeds via two strategic pathways—post-pyrolysis impregnation and in situ co-pyrolysis—each offering distinct advantages for tailoring composite properties to TAD risk-control applications. In post-pyrolysis impregnation, pre-synthesized biochar is saturated with a nano-CaO_2_ suspension followed by calcination, integrating peroxide species into the carbon matrix [13]. This yields a uniform CaO_2_ nanoparticle distribution on the biochar surfaces and within pore structures, enhancing ROS generation and alkaline buffering capacity while maintaining the high surface area and porosity of the parent biochar [50]. For TAD applications, this configuration provides rapid access to reactive CaO_2_ surfaces for inhibitor oxidation while the underlying carbon matrix continues adsorption and microbial habitat functions, creating synergistic risk control [17]. In co-pyrolysis, raw biomass homogenized with nano-CaO_2_ undergoes direct thermal treatment, with nano-CaO_2_ serving as a catalytic agent during pyrolysis [51]. This in situ approach promotes dehydration, enhances devolatilization, and facilitates CO_2_ capture via carbonation reactions, reducing tar formation while increasing biochar yield [46]. According to Wang et al. [51], nano-CaO_2_ undergoes tar reforming and water–gas shift reactions during co-pyrolysis, yielding calcium species that are chemically bonded throughout the carbon matrix rather than merely surface-coated. This structural integration produces superior properties: a stable porous network supporting microbial colonization and contaminant adsorption, combined with oxidative and alkaline reactivity distributed throughout the material for sustained inhibitor mitigation [13,14,20,51]. For TAD applications, the integrated structure offers enhanced durability against thermophilic physicochemical stresses, with calcium species being less susceptible to leaching than surface coatings [14]. Table 4 presents key reactions during nano-CaO_2_-catalyzed biomass pyrolysis with thermodynamic data and their relevance to risk mitigation.

During pyrolysis, nano-CaO_2_ decomposes endothermically, releasing oxygen that cracks heavy tar molecules while providing thermal energy for reforming reactions. Zhang et al. [52] demonstrated that perovskite oxygen carriers achieved 88.7% tar removal efficiency in biomass gasification, maintaining >88.0% efficiency after 10 redox cycles. The resulting quicklime performs dual functions: catalyzing steam–carbon reactions to produce hydrogen-rich syngas and capturing CO_2_ via carbonation to CaCO_3_ [51]. This self-enhancing cycle purifies syngas, increases hydrogen yield, and generates a nano-CaO_2_/BC composite with regenerable properties (via CaCO_3_ calcination) suitable for catalysis and risk-controlled TAD enhancement.

### 4.3. Physicochemical Properties of Nano-CaO_2_/BC: Engineered for Comprehensive Risk Mitigation

The functionalization of biochar with nano-CaO_2_ yields an engineered composite with superior physicochemical properties, tailored to address the operational risks and environmental hazards associated with tofu TWW treatment. This composite enhances process stability, improves nutrient retention, and mitigates risks related to process failure, pollutant diffusion, and resource inefficiency. The system generates two primary valorization streams: biogas, a renewable energy source suitable for heat and power generation or upgrading to biomethane, and a nutrient-enriched digestate that serves as a high-performance soil amendment. By returning this digestate to agricultural systems, the approach supports a closed-loop circular economy, reduces reliance on synthetic fertilizers, and strengthens agricultural resilience. The key characteristics of the nano-CaO_2_/BC composite are examined in detail in the following sections.

### 4.4. pH and Functional Groups: Acidification Risk Control

Nano-CaO_2_/BC introduces multi-mechanistic enhancement to TAD of TWW, systematically addressing process-failure risks intensified under elevated-temperature operation. Slow hydrolysis of nano-CaO_2_ provides continuous alkalinity release, stabilizing pH within methanogenic optima against VFA accumulation [14,17,49]. Unlike single-dose amendments, sustained release maintains protection throughout digestion cycles, counteracting the widened kinetic mismatch between acidogenesis and methanogenesis under thermophilic conditions [50]. Concurrent H_2_O_2_ decomposition generates ROS and transient micro-aerobic zones, facilitating partial oxidation of accumulated VFAs before methanogenic inhibition occurs, directly addressing the acidification root cause [15,53,54]. This provides alternative electron sinks under TAD, where accelerated acidogenesis rapidly overwhelms methanogenic capacity. The biochar matrix serves as a conductive scaffold promoting direct interspecies electron transfer (DIET) between syntrophic bacteria and methanogens, enhancing the thermodynamic efficiency of syntrophic VFA oxidation (propionate, butyrate conversion to acetate) [14]. This directly addresses propionate accumulation, a common thermophilic process-failure precursor, by accelerating rate-limiting acetogenesis.

Nano-CaO_2_ modification induces surface oxidation, generating oxygen-containing functional groups (carboxyl, hydroxyl, carbonyl) that increase surface polarity and negative charge density [13,51]. Enhanced cation exchange capacity retains NH_4_^+^ and cationic nutrients, reducing free NH_3_ inhibition risk while increasing bioavailability for methanogenic consortia [20]. Augmented adsorptive affinity mitigates inhibitory compounds, while providing a colonizable surface for robust biofilm development, stabilizing the microbial community under TAD [19,54]. Synergistic effects combining sustained buffering, oxidative VFA consumption, DIET enhancement, nutrient retention, and inhibitor adsorption accelerate organic matter breakdown, stabilize the TAD process, and increase biogas yields while systematically reducing process-failure probability.

### 4.5. Porosity and Surface Area: Adsorption and Habitat Enhancement

Nano-CaO_2_ functions as a structural porogen during biochar synthesis, fundamentally enhancing textural properties critical for TAD risk control [14]. Thermal decomposition generates gaseous oxygen micro-bubbles within the carbon matrix, preventing pore collapse and adjacent structure fusion during pyrolysis [15,16]. This creates a hierarchical pore network substantially increasing specific surface area and total pore volume [21]. The resulting architecture, rich in micropores and mesopores, provides an extensive accessible surface for the adsorption of inhibitory compounds from digested sludge, directly mitigating inhibitor-accumulation risks that destabilize TAD performance [55]. For TAD applications, enhanced porosity serves multiple risk-control functions simultaneously. Expanded surface area provides abundant sites for VFA adsorption, reducing liquid-phase concentrations and mitigating acidification risk before methanogenic consumption occurs. Micropores selectively retain ammonia through size exclusion and surface interactions, reducing bioavailable NH_3_-N and mitigating ammonia inhibition risk intensified under thermophilic conditions [19]. Mesopores provide accessible pathways for microbial colonization, enabling biofilm development within protected niches where microorganisms are shielded from shear stress and toxic compounds while maintaining substrate access [14]. This protected colonization is particularly valuable under TAD, where reduced microbial diversity increases the importance of maintaining active populations of key functional groups.

The nanoporous structure serves as an ideal micro-habitat for anaerobic consortia, facilitating substrate–enzyme interactions that enhance metabolic rates and accelerate organic matter conversion to methane [20]. Enhanced porosity directly correlates with improved catalytic activity by exposing more active sites and promoting mass transfer, reducing the hydraulic retention time required for complete stabilization [51]. In thermophilic systems, where elevated temperatures accelerate both desirable metabolic reactions and potentially damaging stress responses, protected microenvironments help maintain microbial activity during transient perturbations [46]. Upon land application as digestate, this engineered porosity remains functionally relevant, improving soil water-holding capacity, aeration, and providing a high-surface-area substrate for nutrient retention and microbial habitat [56]. The calcium-rich pore structure contributes to soil aggregation through cation bridging between organic matter and mineral particles, forming stable macroaggregates that resist erosion and improve root penetration [22,28]. This translates initial material enhancement into sustained agronomic benefits, addressing soil degradation risks while closing the nutrient loop and transforming digestate from waste product into a valuable soil amendment for agricultural sustainability.

## 5. Roles of Nano-CaO_2_/BC in Enhancing Thermophilic Anaerobic Digestion of Tofu Wastewater: A Quantitative Risk-Mitigation Analysis

The TAD in TWW is fundamentally constrained by amplified process risks: rapid acidification from accelerated carbohydrate hydrolysis and NH_3_ inhibition intensified by temperature dependent NH_3_/NH_4_^+^ equilibrium shifts, H_2_S toxicity from enhanced sulfate reduction, and recalcitrant compound accumulation—each exacerbated under elevated-temperature operation [5,11]. Nano-CaO_2_/BC constitutes a rationally designed multifunctional amendment that orchestrates a self-regulating mechanism to synergistically address these metabolic imbalances through integrated risk control [13,14,19]. The mechanism synergistically integrates BC’s adsorptive properties and microbial-habitat provision with nano-CaO_2_’s controlled oxidative and alkaline potential to orchestrate dynamic microenvironments within thermophilic digesters [18,20,57]. This integration achieves: (i) sustained pH buffering via slow Ca(OH)_2_ release, counteracting VFA accumulation [17,58]; (ii) controlled micro-aeration through H_2_O_2_ decomposition, generating ROS that oxidize accumulated VFAs [15], H_2_S [19], and recalcitrant compounds while providing alternative electron sinks [20]; (iii) adsorptive mitigation of inhibitory NH_3_-N and sulfide via the porous carbon matrix, reducing bioavailable concentrations below toxicity thresholds [54,55]; and (iv) enhanced direct interspecies electron transfer through conductive biochar scaffolding, accelerating syntrophic VFA oxidation [14]. This coordinated action systematically addresses the kinetic imbalances in conventional TAD of TWW, mitigating risks within a unified risk-management framework. It transforms process-stabilized organic conversion into improved bioenergy recovery and nutrient-rich digestate valorization.

### 5.1. Failure Mode and Effects Analysis: Semi-Quantitative Projection of Risk Reduction

To evaluate the potential risk-mitigation efficacy of nano-CaO_2_/BC in TAD of TWW, failure mode and effects analysis is employed as a semi-quantitative theoretical projection tool [26]. It should be emphasized that the FMEA scores presented herein are not derived from a specific TWW-TAD case study with nano-CaO_2_/BC, as no such experimental data currently exist. Rather, the scores represent systematic extrapolations based on mechanistic understanding from analogous systems (activated sludge, food waste, and manure digestion with CaO_2_, Ca(OH)_2_, and modified biochars) as documented in the reviewed literature. The analysis serves to illustrate the conceptual risk-reduction potential and guide future experimental design, not to claim validated operational performance. FMEA evaluates each potential failure mode according to three dimensions: Severity (S), representing the impact of failure on system performance (scale 1–10, with 10 representing catastrophic failure); Occurrence (O), representing the probability of failure under normal operating conditions (scale 1–10, with 10 representing near-certain occurrence); and Detection (D), representing the likelihood that failure would be detected before causing significant damage (scale 1–10, with 10 representing near-impossible detection) [59]. The risk priority number (RPN) is calculated as the product of these three factors (RPN = S × O × D), providing a quantitative basis for prioritizing risk-mitigation efforts [26,59].

The scoring criteria adopted are as follows: Severity is based on methane yield reduction (S = 9: >80% reduction, complete failure; S = 8: 60–80% reduction; S = 7: 40–60% reduction; S = 6: 20–40% reduction; S = 5: 10–20% reduction; S = 4: 5–10% reduction). Occurrence is based on probability estimation from comparable substrates (O = 8: >70% probability within one hydraulic retention time; O = 7: 50–70%; O = 6: 30–50%; O = 5: 10–30%; O = 3: 1–10%; O = 2: <1%). Detection is based on monitoring capability (D = 9: detectable only through off-line analysis with >24 h delay; D = 8: detectable through off-line analysis within 12–24 h; D = 7: detectable through on-line sensors with >4 h delay; D = 5: detectable through on-line sensors within 1–4 h; D = 4: detectable through real-time on-line sensors). These criteria were developed based on standard FMEA practice in wastewater-treatment applications and adapted to TAD-specific failure modes using mechanistic knowledge from the reviewed literature [5,11,26,59]. The scoring was performed by the authors through iterative consensus building based on the evidence synthesis presented in Section 3 and Section 4. It is acknowledged that formal expert elicitation would improve the reliability of the scores; this is identified as a priority for future research.

Table 5 and Table 6 present the FMEA analyses for conventional and nano-enhanced TAD of TWW, respectively. For conventional TAD (Table 5), acidification failure emerges as the dominant risk with an RPN of 504, reflecting high severity (complete process failure requiring reactor restart), high occurrence probability (given TWW’s high carbohydrate content and thermophilic kinetics), and moderate detection difficulty (pH monitoring provides an early warning, but intervention windows are short) [11]. Ammonia inhibition yields an RPN of 384, with high severity (significant reduction in CH_4_ yield), moderate occurrence probability (dependent on protein content and temperature), and poor detection (early-stage ammonia stress is difficult to distinguish from other inhibitors) [5]. H_2_S toxicity (RPN of 315) and VFA accumulation (RPN 336) represent additional significant risks that require active management [30,39]. With nano-CaO_2_/BC intervention (Table 6), all risk dimensions are projected to show substantial improvement based on mechanistic evidence from analogous systems [13,14,17,19]. For acidification failure, the severity score is maintained at 9 because a catastrophic pH collapse, should it occur, would still cause complete process failure regardless of the additive’s presence [59]. The risk reduction is therefore driven exclusively by a drastic decrease in occurrence probability owing to sustained alkaline buffering, micro-aerobic VFA oxidation, and enhanced DIET, coupled with improved detection through more stable pH profiles [20,54,60]. This yields a projected RPN of 72, corresponding to an 85.7% reduction.

The NH_3_ inhibition RPN is projected to decrease by 87.0% to 50, reflecting the combined effects of ammonium adsorption on biochar surfaces (87.83 mg/g for Mg/Fe-BC) [20,45], the equilibrium shift toward ionized NH_4_^+^ at elevated pH [17], and the potential oxidation of ammonia by ROS as demonstrated in aquaculture wastewater [54]. The H_2_S toxicity RPN is projected to decrease by 89.8% to 32, driven by direct oxidation of sulfide by ROS (80.5% H_2_S reduction at 0.25 g CaO_2_/g VSS) [19], precipitation as calcium sulfide (thermodynamically spontaneous, ΔG = −170 kJ/mol) [53], and suppression of sulfate-reducing bacteria activity through micro-aeration [15]. The VFA accumulation RPN is projected to decrease by 82.1% to 60, reflecting the combined effects of enhanced syntrophic consumption through DIET [14], micro-aerobic oxidation [16], and adsorption on BC surfaces [13]. In addition to these in situ mitigation pathways, VFA recovery from fermentation broth for downstream valorization represents a complementary circular economy strategy; recent advances in extraction technologies could be integrated with nano-CaO_2_/BC-enhanced TAD for comprehensive resource recovery [61]. Pathogen survival and heavy metal mobilization risks are projected to decrease by 73.3% and 71.4%, respectively, reflecting the enhanced sanitation of thermophilic operations combined with the adsorptive capture of metals by the biochar matrix [35,37,56].

It should be reiterated that these RPN reductions are theoretical projections based on a mechanistic understanding from analogous systems and not on empirically validated results for TWW-TAD with nano-CaO_2_/BC. The FMEA methodology employed expert independent scoring based on a synthesis of evidence from the literature rather than a formal expert elicitation process with calibrated scales, representing a methodological limitation. Future research should validate these projections through (i) formal expert panel assessments using the Delphi methodology, (ii) pilot-scale experimental determination of failure-mode frequencies, and (iii) sensitivity analysis of RPN to scoring assumptions. Nevertheless, the consistent trends across all failure modes, grounded in established mechanistic principles, provide a reasonable qualitative indication of the comprehensive risk mitigation achievable through the nano-CaO_2_/BC intervention.

### 5.2. Mechanistic Analysis of Risk-Mitigation Pathways

At the core of this risk control strategy lies the controlled hydrolysis of nano-CaO_2_, which under thermophilic conditions facilitates sustained ROS release rather than instantaneous decomposition. This temporal regulation aligns ROS generation with the accelerated kinetics characteristic of elevated-temperature anaerobic digestion [14]. The generated H_2_O_2_ subsequently decomposes (either catalytically on the biochar surface or through microbial pathways) to produce molecular oxygen [13,50]. This slow-release oxygenation establishes transient micro-aerobic niches within the predominantly anaerobic bulk matrix, creating localized oxygen gradients that support spatially organized microbial consortia [15]. Under thermophilic regimes, reduced oxygen solubility paradoxically enhances this micro-aeration effect by limiting oxygen penetration depth and maintaining steep concentration gradients that avert complete system aeration [16]. These micro-aerobic zones fulfill a dual risk mitigation function. Primarily, they selectively stimulate facultative hydrolytic and acidogenic bacteria, accelerating the depolymerization of complex macromolecules in TWW into readily biodegradable monomers. This addresses the hydrolysis limitation that otherwise prolongs retention times and increases volumetric reactor demands. Rashvanlou et al. [15] demonstrated that micro-aerobic pretreatment significantly enhances anaerobic digestion: a 40 h pretreatment at optimized airflow achieved a 30.6% reduction in volatile suspended solids alongside a 65.1% increase in soluble COD. Microbial activity surged by 597% and 170% after 20 and 30 h, respectively, with flow cytometry confirming 67.9% cell viability at 40 h (approximately 40% higher than controls), culminating in a 221% increase in cumulative methane production relative to conventional anaerobic digestion. This enhancement represents concurrent process acceleration (mitigating retention time risk) and yield improvement (addressing resource waste risk) [16]. 

Concurrently, H_2_O_2_ and its derivative ROS (particularly hydroxyl radicals (•OH) generated via Fenton-like reactions catalyzed by iron impurities in biochar or thermophilic sludge) initiate mild advanced oxidation within the sludge matrix [62]. This micro-oxidative pretreatment transforms recalcitrant organic matter and, critically, oxidizes accumulated VFAs, thereby directly alleviating the primary driver of acidification and reactor destabilization [11]. Batch optimization identified 10 g/L CaO_2_ as optimal for VFA degradation, while long-term reactor operation confirmed that calcium carbonate precipitation substantially reduced VFA concentrations in sludge granules, illustrating the synergistic chemical–biological VFA control mechanism [63]. Liu et al. [62] reported that activated carbon combined with calcium peroxide (AC/CaO_2_) achieved the highest specific methane yield from food waste (434.4 mL/g VS), outperforming Fe_3_O_4_/CaO_2_ and individual additives, attributable to enhanced ROS generation, improved hydrolytic acidification, and the formation of robust microbial aggregates with elevated enzymatic defense and damage repair capacity. Under TAD conditions (where oxidative stress imposes greater physiological demands), the BC matrix exerts critical control by adsorbing excess radicals and confining oxidative activity to defined zones [13]. Calcium peroxide pretreatment has been shown to enhance AD via radical-mediated oxidation [48].

The co-generated Ca(OH)_2_ from CaO_2_ hydrolysis provides robust, sustained alkaline buffering that directly counteracts acidification risk [17]. This neutralizes acidic fermentation intermediates, mitigates product inhibition, and maintains pH optima for thermophilic methanogenic archaea. Junoh et al. [49] optimized Ca(OH)_2_ pretreatment for food waste using response surface methodology (166.98 mEq/L, 6.1 g/L, 1 h), achieving 864.19 mL CH_4_/g VS destroyed (a 20.0% increase over untreated controls). Ji et al. [64] further demonstrated that combined 1.0% Ca(OH)_2_ and steam explosion (1.5 MPa) pretreatment of corn stover increased cumulative CH_4_ yield by 61.5% and enhanced biodegradability from 43.0% to 69.5%, confirming that alkaline treatment synergizes with physical disruption to improve substrate accessibility, a principle directly applicable to the particulate organic fraction of TWW.

Khor et al. [58] identified optimal Ca(OH)_2_ pretreatment for grass at 7.5% lime loading, increasing methane yield by 37.3%; post-extrusion Ca(OH)_2_ treatment yielded additional increases of 15.2% (grass), 11.2% (maize straw), and 8.2% (sprout stem), with corresponding COD conversion improvements of 10.3%, 9.0%, and 6.8%, respectively. These findings confirm that calcium-based alkaline treatment enhances both the rate and extent of organic conversion, addressing process efficiency dimensions of resource waste risk. Yang et al. [55] co-digested Ca(OH)_2_-pretreated levulinic acid wastewater with corn stalk (0.31 g/L sulfate loading, 32.3 g/L substrate), achieving 249.93 mL CH_4_/g VS (substantially exceeding mono-digestion (182.53 mL/g VS)) with 98.1% sulfate removal, demonstrating simultaneous enhancement of methanogenesis and suppression of sulfate reduction, thereby addressing both energy recovery and H_2_S toxicity risks. Yang et al. [56] further reported that Ca(OH)_2_ addition increased biogas yield to 372.2 mL/g VS (61.7% over control), with high-concentration Ca(OH)_2_ achieving 422.8 mL/g VS (83.7% increase). Critically, the high-Ca(OH)_2_ digestate increased ryegrass biomass by 19.7% while reducing plant Zn and Cu concentrations by 31.1% and 39.3%, respectively, confirming that risk control benefits extend beyond the digester to agricultural end-use [56,64]. The coordinated integration of micro-aerobic pretreatment, in situ advanced oxidation, and alkaline stabilization fundamentally rectifies carbon metabolic pathways, preventing intermediate accumulation that compromises conventional TAD of TWW. As projected in the FMEA, this approach could reduce RPN for carbon-flow-related failures by 80–90% (pending experimental validation).

### 5.3. Sulfur, Nitrogen, and Phosphorus Risk-Control Pathways

Beyond carbon metabolism, nano-CaO_2_/BC fundamentally governs the fate of sulfur, nitrogen, and phosphorus—elements central to the multidimensional risks inherent in TWW treatment [17,55]. Mitigation of H_2_S, a corrosive and toxic gas posing both process inhibition and infrastructure degradation risks, occurs through complementary thermodynamically driven pathways under thermophilic conditions [17]. The oxidative potential of nano-CaO_2_, mediated by H_2_O_2_ decomposition and ROS generation, directly oxidizes sulfide species (S^2−^/HS^−^) to elemental sulfur or sulfate, thereby depleting the bioavailable toxicant pool [14,19,20]. Simultaneously, the alkaline environment imparted by Ca(OH)_2_ shifts the aqueous sulfide equilibrium toward the less volatile HS^−^ ion, reducing H_2_S partial pressure in biogas and minimizing downstream corrosion [55]. According to Guo et al. [53], released Ca^2+^ precipitates sulfide as insoluble calcium sulfide (CaS). In the strongly reducing thermophilic digester milieu (Eh < −200 mV), thermodynamics favor sulfide (S^2−^) over sulfate as the dominant species. Ca^2+^ precipitation with abundant S^2−^ to form CaS is spontaneous (ΔG = −170 kJ/mol), ensuring rapid and quantitative sulfide removal. Conversely, oxidation of S^2−^ to SO_4_^2−^ (a prerequisite for CaSO_4_ formation) is non-spontaneous (positive ΔG) under these reducing conditions [53]. Thus, thermodynamics selectively drive CaS precipitation, precluding acidification from CaSO_4_ hydrolysis and sequestering sulfur as a stable mineral phase that reports to the digestate rather than partitioning to biogas.

**Figure 4:**
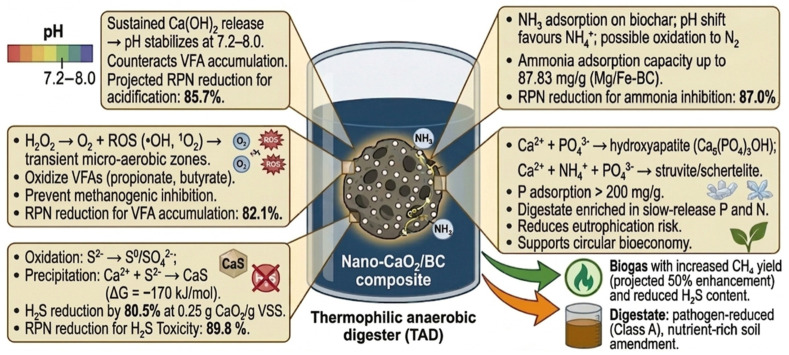
Schematic representation of nano-calcium-peroxide-modified biochar as an additive in thermophilic anaerobic digestion of tofu wastewater.

Xu et al. [19] demonstrated that CaO_2_ fundamentally reconfigures anaerobic sulfur distribution. At 0.25 g/g VSS, H_2_S production declined from 314.6 to 61.3 (×10^−4^ mg/g VSS), an 80.5% reduction, while short-chain fatty acids increased to 266.5 mg COD/g VSS and hydrogen yield rose to 10.2 mL/g VSS. The mechanism involves the radical-mediated (•OH and •O_2_^−^) degradation of extracellular polymeric substances, reducing α-helix, β-sheet, and carbonyl contents by 23.2%, 19.7%, and 24%, respectively. This disintegration released metal ions that precipitated dissolved sulfides as metal sulfides, further suppressing H_2_S. Subsequent radical attack induced cell lysis (viable cells reduced from 90.7% to 81.4%) and inhibited sulfate-reducing microorganisms, decreasing activities of key enzymes (adenosine-5′-phosphosulfate reductase and methionine lyase by 64.8% and 41.3%, respectively) [19]. This comprehensive suppression of sulfate reduction disrupts competitive hydrogen and acetate consumption, directly enhancing methane yield while controlling H_2_S toxicity. Under TAD, where sulfate reduction kinetics are accelerated, this inhibition is critical for preserving methanogenic dominance. The schematic representation of nano Ca_2_O_2_/BC as an additive in TAD of TWW is presented in Figure 4.

The enhanced phosphorus adsorption capacity of nano-CaO_2_/BC derives from its engineered mineral composition, which mitigates pollution diffusion risks (particularly eutrophication) while addressing resource waste through nutrient recovery [13,22,28]. Calcination during BC production and subsequent nano-CaO_2_ hydrolysis enrich the composite with Ca(OH)_2_ and CaCO_3_ [46,51]. In the thermophilic digester aqueous environment, these compounds dissociate, elevating free Ca^2+^ concentrations [17]. These ions rapidly interact with orthophosphate (PO_4_^3−^) to form stable hydroxyapatite (Ca_5_(PO_4_)_3_(OH)) precipitates [21,28]. This reaction removes phosphorus from the liquid phase, reducing discharge concentrations and eutrophication potential, while sequestering phosphorus in the digestate as a slow-release fertilizer, transforming a waste risk into an agricultural resource [28].

Li et al. [13] synthesized a nano-CaO_2_/BC composite achieving phosphorus adsorption following pseudo-second-order kinetics and Langmuir–Freundlich isotherm behavior, with a maximum capacity of 213.22 ± 13.57 mg g^−1^. Precipitation dominated the mechanism, and the phosphorus-laden product promoted seedling growth, confirming soil amendment potential. This capacity (21–23% phosphorus by weight) substantially addresses the 0.97–95.50 mg/L phosphorus concentrations typical of TWW [4]. Xu et al. [22] reported calcium-modified BC achieved 168.2 mg/g adsorption (27.6-fold greater than unmodified precursor) across pH 4–12. Tang et al. [21] demonstrated that CaO addition during sewage sludge pyrolysis promoted phosphorus transformation to hydroxyapatite, reaching 25 wt% of total phosphorus in BC, suggesting that nano-CaO_2_/BC not only captures phosphorus during digestion but stabilizes it in plant-available forms for soil application. Lee et al. [28] showed that calcined mussel shells transformed to CaO and Ca(OH)_2_, achieving 66.70 mg/g Langmuir capacity via Ca_5_(PO_4_)_3_(OH) precipitation, with phosphorus-laden material (43.07% calcium-bound phosphorus, 55.64% residual phosphorus) promoting plant growth at 0.73 g/kg soil, confirming that calcium-mediated phosphorus capture yields digestate with genuine agronomic value.

Concurrently, nano-CaO_2_/BC optimizes nitrogen balance, addressing both ammonia inhibition and nitrogen loss risks. The sustained alkaline environment from Ca(OH)_2_ shifts the NH_4_^+^-NH_3_ equilibrium toward NH_3_ [17,18], facilitating stripping and recovery—transformable to ammonium sulfate fertilizer via acid scrubbing. Fixed nitrogen retained in digestate, combined with precipitated phosphorus, converts organic residue into nutrient-enriched soil amendment [28]. Nano-CaO_2_/BC promotes nitrogen fixation through chemisorption and precipitation: Ca^2+^ reacts with phosphate and NH_4_^+^ to form minerals such as schertelite ((NH_4_)_2_Ca(HPO_4_)_2_·2H_2_O) or co-precipitates within magnesium phosphate complexes as struvite (MgNH_4_PO_4_·6H_2_O), immobilizing nitrogen in slow-release form [13,17]. Under TAD, Ca_5_(PO_4_)_3_(OH) precipitation is thermodynamically favored over ammonium calcium phosphates [22]; however, aqueous ammonium influences hydroxyapatite crystallization kinetics and morphology, with some ammonium incorporated via surface adsorption or co-precipitation, contributing to solid-phase nitrogen retention. Struvite formation, critically dependent on magnesium availability, is optimized by the alkaline environment (pH 8.5–9.0) that favors PO_4_^3−^ speciation [13].

Nevertheless, high calcium concentrations favor Ca_5_(PO_4_)_3_(OH) over MgNH_4_PO_4_·6H_2_O, creating competitive dynamics requiring management through dosing strategies to achieve desired nutrient recovery outcomes [60]. Zhao et al. [20] engineered a CaO_2_-based composite achieving >70% NH_4_^+^-N removal from aquaculture wastewater via slow H_2_O_2_ release, generating singlet oxygen (^1^O_2_) as the dominant ROS to selectively convert NH_4_^+^ to N_2_. Continuous operation maintained 80–99% NH_4_^+^-N and 70–99% antibiotic removal. Koyama et al. [17] demonstrated that Ca(OH)_2_ enhanced ammonia recovery by 50–69% over controls. The UV/persulfate-CaO system completely oxidized 30 mg N/L NH_3_-N within 15 min with 95.0% N_2_ selectivity, via CaO-mediated NH_4_^+^-to-NH_3_ conversion followed by hydroxyl radical oxidation [54]. Thus, nano-CaO_2_/BC functions not as a passive additive but as an integrated, self-regulating micro-ecosystem within TAD, simultaneously controlling phosphorus discharge, recovering nutrients as value-added fertilizer components, and mitigating nitrogen inhibition through coupled physicochemical and biological mechanisms. The substantial enhancement conferred by calcium modification is evident when comparing nano-CaO_2_/BC (213 mg PO_4_^3−^/g) [13] with unmodified biochars: for instance, biochar derived from cyanobacterial biomass achieved a maximum phosphate adsorption capacity of only 5.51 mg/g through monolayer chemisorption [65], underscoring the critical role of calcium-mediated precipitation in achieving high-capacity phosphorus capture.

### 5.4. Comparison with Alternative Enhancement Strategies

To contextualize the proposed technology, a comparative table (Table 7) has been added, evaluating nano-CaO_2_/BC [13,14,17,19,20,54,62] alongside conventional Ca(OH)_2_ pretreatment [22,49,58,64], trace element supplementation [66], pristine biochar [12,67], Fe_3_O_4_-modified biochar [47], Mg/Fe-modified biochar [45], KOH-activated biochar [68], FeCl_3_-impregnated biochar [69], metal-engineered biochar [70], nZVI/biochar, and MnO_2_-modified biochar [69,71]. The table summarizes key mechanisms, reported methane enhancement, and the capacities for ammonia, H_2_S, pH buffering, and nutrient recovery. The comprehensive and technical comparison of TAD enhancement strategies for high-strength organic waste is shown in Table 7.

The data in Table 7 reveal distinct functional profiles. Conventional Ca(OH)_2_ pretreatment enhances methane yield by 15–84% through alkaline hydrolysis and partial solubilization of organic matter, yet it provides no inherent ammonia or sulfide control and offers only transient pH buffering [22,49,58,64]. Trace element supplementation (Co, Ni, Se, Mo, W) achieves 30–65% improvement in methane production by supplying essential metallo-cofactors that accelerate VFA metabolism, but it lacks direct inhibitor adsorption, pH stabilization, or nutrient recovery capacity [66]. Pristine BC increases methane yield by 18–37% via physical adsorption of inhibitors, DIET, and mild pH buffering through surface functional groups; however, its NH_3_ adsorption capacity is limited and it offers no specific H_2_S oxidation pathway [12,67]. Iron-based modifications address some of these limitations. Fe_3_O_4_-modified biochar enhances DIET (25–56% methane increase) but does not directly mitigate H_2_S or provide pH buffering [47]. Mg/Fe-modified biochar stands out for its high NH_4_^+^-N adsorption capacity and potential for struvite precipitation, yielding a remarkable 116–229% methane increases in chicken manure digestion, though its H_2_S control remains low [45]. Similarly, KOH-activated biochar accelerates methane production rate by 52% through ultra-high surface area and enhanced electron transfer, but it does not address sulfur toxicity or nutrient recovery [68]. FeCl_3_-impregnated biochar promotes diverse methanogenic pathways (22.5% methane increase) but lacks quantitative data on NH_3_ and H_2_S control [69].

Metal-engineered biochar loaded with Fe, Ni, Co, and Mn reduces the lag phase from 22.3 to 3.7 days and achieves 224.7 NmL CH_4_/g VS by creating spatial niches for DIET, yet its performance against NH_3_ and H_2_S inhibition is not reported [70]. Nano-zero-valent iron/biochar (nZVI/BC) provides the best H_2_S control among single additives, reducing H_2_S from 8.32 to 0.22 mL through FeS precipitation, and improves methane by 29.2–46.7% [69]. MnO_2_-modified biochar offers moderate improvement under high NH_3_ stress (12.7% methane increase) but lacks direct H_2_S mitigation [71]. In contrast, nano-CaO_2_/BC is projected to combine sustained pH buffering (slow Ca(OH)_2_ release), oxidative H_2_S and VFA control (ROS generation), high NH_4_^+^ adsorption and oxidation, and exceptional phosphorus recovery (>200 mg PO_4_^3−^/g) through calcium phosphate precipitation [13,14,17,19,20,60,62]. While several individual strategies excel in one or two risk dimensions, none provide the integrated multi-risk mitigation that is essential for stabilizing the thermophilic digestion of TWW, where acidification, NH_3_ toxicity, and H_2_S inhibition co-occur [2,4,5,11]. Direct comparative studies under identical TAD-TWW conditions are entirely lacking, and the projected superior performance of nano-CaO_2_/BC must be validated experimentally.

## 6. Economic Feasibility Considerations

Integrating nano-CaO_2_/BC into TAD of TWW can enhance methanogenic yield and process stability, but economic viability must be assessed by balancing the value of extra biogas and improved digestate against the incremental cost of the composite material and dosing equipment [46]. A realistic break-even analysis can be performed on a per-cubic-meter basis using documented market prices and stoichiometric methane yields, avoiding unreliable national-scale extrapolations. The additive cost is governed by the required dosage. Based on the optimized dosage of 6.67 kg/m^3^ reported for BC-enhanced AD [67] and assuming a commercial BC price of 0.08–0.12 USD/kg [72], the material cost of the BC matrix alone amounts to 0.53–0.80 USD per m^3^ of TWW. Loading with nano-CaO_2_ (10–20 wt%) is estimated to add roughly 0.20–0.50 USD per m^3^, bringing the total additive cost to approximately 0.73–1.30 USD/m^3^. These figures may decrease with process optimization and economies of scale.

On the revenue side, the energy value of the additional methane must offset this cost. A typical TWW with a COD of 6000 mg/L (6 kg COD/m^3^) can theoretically yield 0.35 m^3^ CH_4_ per kg COD removed, giving a maximum of 2.1 m^3^ CH_4_/m^3^ [5]. At a gas price of 0.3686 USD/m^3^ (based on electricity generation) [12], the gross energy revenue from full COD conversion is 0.77 USD/m^3^. Thus, even complete conversion of all COD cannot cover the high end of the additive cost (1.30 USD/m^3^) if the additive merely serves as a catalyst without drastically increasing the fraction of COD converted. However, if nano-CaO_2_/BC improves the conversion efficiency from, say, 50% to 80%, the incremental methane would be (0.80 − 0.50) × 2.1 = 0.63 m^3^ CH_4_/m^3^, worth about 0.23 USD/m^3^—which is still insufficient. This simple calculation demonstrates that the direct energy revenue alone is unlikely to produce a net profit unless the additive cost is substantially lowered and/or substantial co-benefits are monetized. 

The primary economic drivers are therefore the risk avoidance benefits (reduced reactor downtime, avoided environmental penalties, lower maintenance costs) and the fertilizer value of the digestate. When these are included conservatively estimated at 0.10–0.30 USD/m^3^ for risk avoidance and 0.05–0.15 USD/m^3^ for nutrient value, the total benefit per m^3^ can reach approximately 0.38–0.68 USD. At the lower additive cost (0.73 USD/m^3^) and under optimistic performance, a small positive margin becomes possible, but negative net returns remain likely under current cost structures. Therefore, economic feasibility hinges on achieving sufficient process intensification to drastically reduce additive dosage, exploring cheaper local feedstocks, and developing revenue mechanisms for digestate and avoided emissions. The analysis underlines the need for pilot-scale data to refine the key parameters (actual methane enhancement, additive lifetime, and operational savings) before any large-scale investment can be justified. Ultimately, a rigorous life-cycle costing that internalizes environmental externalities is required to fully capture the value of the risk-controlled circular bioeconomy model.

## 7. Challenges, Future Perspectives, and Risk-Management Optimization

Notwithstanding the projected efficacy of nano-CaO_2_/BC in enhancing TAD and mitigating multidimensional risks associated with TWW treatment, industrial translation remains contingent upon resolving critical research and development challenges. The principal obstacle is the absence of standardized synthesis protocols; interplay among feedstock selection, pyrolysis conditions [18,51], and nano-CaO_2_ loading dictates hierarchical pore architecture and calcium–carbon interface stability, producing variable risk control performance [13]. To address this, we propose the following preliminary optimal synthesis matrix based on the physicochemical data in Table 3 and mechanistic requirements:

1. Feedstock: Soybean stover or peanut shells, selected for (i) high surface-area development at 700 °C (420–448 m^2^/g), (ii) alignment with circular economy by valorizing tofu production residues, and (iii) favorable ash composition supporting alkaline functionality.
2. Pyrolysis temperature: 700 °C, to maximize surface area (>400 m^2^/g target), ensure structural stability under thermophilic conditions, and develop alkaline pH (>10.5).
3. Nano-CaO_2_ loading: 10–20% *w*/*w* via post-pyrolysis impregnation, providing sufficient reactive capacity for pH buffering and ROS generation while preserving BC pore structure.
4. Key performance indicators (KPIs): Specific surface area >400 m^2^/g (BET), calcium content 10–20% *w*/*w*, alkaline buffering capacity >5 meq/g, and H_2_O_2_ release rate 0.5–2.0 mM/day at 55 °C.

Industry standards specifying these KPIs would address synthesis uncertainty and facilitate technology adoption. The long-term fate of the composite within continuous TAD systems remains ambiguous. Surface passivation, active-phase dissolution, and pore occlusion from CaCO_3_ precipitation may progressively diminish catalytic and buffering capacity [14]. Under thermophilic conditions, elevated temperatures accelerate both beneficial reactions and deleterious processes: mineral dissolution, carbon oxidation, and biofilm overgrowth that masks active sites [19]. Specific mechanisms requiring investigation include CaO_2_ consumption kinetics (half-life ~15–30 days at 55 °C) [50], biochar surface oxidation and potential loss of DIET conductivity, and competitive CaCO_3_ formation consuming active Ca(OH)_2_ [17]. Longitudinal studies tracking performance over multiple retention times are required to establish replacement schedules and life-cycle material requirements.

Operational temperature presents a complex optimization challenge: maintaining metabolic integrity of thermophilic methanogens (optimal 55–60 °C) [27] while regulating kinetic release of alkalinity and micro-oxygen from CaO_2_. Wang et al.’s [73] CaO_2_ hydrolysis and H_2_O_2_ decomposition follow Arrhenius kinetics, with rates approximately doubling per 10 °C increase. At 55 °C, reaction rates are 3–4-fold higher than at 35 °C, accelerating active material consumption and reducing effective residence time [73]. Balancing accelerated consumption against enhanced risk control requirements necessitates optimized initial loading and potentially encapsulated formulations providing sustained release at elevated temperatures [17]. Pathogen inactivation (mediated through elevated pH, ROS generation, and enhanced adsorption) requires explicit validation via metagenomic analysis to ensure biosolid safety [8,20]. Although FMEA indicates substantial pathogen reduction, direct confirmation through culture-based and molecular detection methods is necessary to satisfy regulatory requirements for land application [26,35,37]. Concurrently, the potential for nano-CaO_2_/BC to mobilize or immobilize heavy metals in TWW must be characterized to ensure digestate complies with agricultural quality standards [56]. Regarding large-scale production costs, current commercial BC prices (0.08–0.12 USD/kg) reflect moderate production scale [72]; nano-CaO_2_ synthesis adds roughly 200–500 USD/ton for the nano-CaO_2_ component, but costs are expected to decline with optimization [13,14,17,19,20,54,62]. The sensitivity analysis in Section 6 shows that a 30% cost reduction through synthesis optimization and economies of scale could improve the economic balance. Integration of nano-CaO_2_/BC production with existing biochar facilities at tofu processing plants could further reduce costs through waste heat utilization and feedstock availability.

Concerning nanoparticle fate and ecotoxicological considerations, nano-CaO_2_ undergoes rapid hydrolysis to bulk Ca(OH)_2_ (complete conversion within hours at 55 °C) [50,73], which subsequently carbonates to CaCO_3_. The resulting calcium species are non-nanoparticulate and pose negligible nano-specific toxicological risk to soil microbiomes [22]. Residual ROS from CaO_2_ hydrolysis is short-lived in the strongly reducing thermophilic digester matrix (ORP < −300 mV), with hydroxyl radical half-lives on the order of nanoseconds [73]. However, potential ecotoxicological effects during digestate land application warrant investigation, particularly (i) chronic effects of elevated Ca^2+^ concentrations on soil microbial community structure, (ii) potential accumulation of BC-borne polycyclic aromatic hydrocarbons (PAHs) formed during pyrolysis [74], and (iii) interactions between calcium-rich digestate and soil organic matter dynamics. Regulatory frameworks including the EU Fertilizing Products Regulation (2019/1009) [75] and U.S. EPA Part 503 [76] biosolids standards establish contaminant limits and pathogen-reduction requirements that would govern nano-CaO_2_/BC-enhanced digestate land application, though nano-specific provisions remain under development.

Within a risk governance framework, systematic identification and management of derived risks is essential. Key residual risks include long-term pedospheric accumulation following repeated digestate application, which may elevate soil calcium and pH beyond agronomic optima [28]. Although nano-CaO_2_ rapidly converts to bulk Ca(OH)_2_ and CaCO_3_, the BC fraction’s environmental persistence necessitates long-term field trials to assess cumulative impact [22]. Economic viability faces risks from volatile material costs and supply chain disruptions. A robust management framework should incorporate dynamic risk monitoring, integrating real-time sensor networks with supervisory control and data acquisition for early warning and proactive intervention [46]. Risk thresholds (pH < 6.8, VFAs > 2000 mg/L) can be calibrated to the system’s enhanced buffering capacity. Establishing cross-sector collaborative governance linking producers, TAD operators, regulators, and agricultural end-users is critical for managing land application risks [38]. Such frameworks codify digestate quality standards, implement transparent certification, and optimize application protocols, transforming digestate into a marketable product while ensuring residual environmental and food safety risks are systematically controlled.

To bridge the critical gap between theoretical projection and empirical validation, we propose a three-phase experimental validation framework:

- Phase 1: Biochemical Methane Potential (BMP) Assays. Batch assays at 55 °C using synthetic TWW (formulated based on Table 1 and Table 2) with nano-CaO_2_/BC dosages of 0, 2.5, 5.0, 7.5, 10.0, and 15.0 g/L. Primary endpoints: cumulative methane yield (mL CH_4_/g COD), VFA profiles, pH stability, NH_3_-N and H_2_S concentrations. Duration: 30 days, triplicate reactors.
- Phase 2: Continuous Stirred Tank Reactor (CSTR) Operation. Pilot-scale CSTR (100–500 L working volume) operated at 55 °C, HRT 10–15 days, organic loading rate 2–5 kg COD/m^3^/day, with nano-CaO_2_/BC at optimized dosage from Phase 1. Primary endpoints: steady-state methane productivity (m^3^ CH_4_/m^3^ reactor/day), COD removal efficiency, process stability indicators (pH, VFA/alkalinity ratio), and microbial community analysis via 16S rRNA sequencing. Duration: minimum 3 HRT cycles after steady-state achievement.
- Phase 3: Digestate Quality Assessment. Comprehensive characterization of digestate from Phase 2 including nutrient speciation (N, P, K), heavy metal content, pathogen indicators (fecal coliforms, *Salmonella*, helminth ova), and phytotoxicity (seed germination index). Soil incubation studies to assess nitrogen mineralization rates and phosphorus availability.

This framework, if implemented, would provide the empirical foundation necessary to transform the theoretical projections presented in this review into validated process-engineering parameters.

## 8. Conclusions

The application of nano-CaO_2_/BC in the TAD of TWW represents a conceptual advancement from conventional treatment methods to a precision-engineered, multifunctional remediation strategy. The composite operates through a synergistic mechanism: the controlled release of CaO_2_ provides sustained micro-aeration that hydrolyzes recalcitrant organics and suppresses sulfide formation, while simultaneously generating Ca(OH)_2_ for robust pH buffering against VFA accumulation. The high surface area of the biochar matrix immobilizes inhibitory NH_3_ and H_2_S, supports syntrophic metabolism, and creates a conductive framework for direct interspecies electron transfer. These integrated actions are projected to enhance the metabolic efficiency and resilience of thermophilic methanogenic consortia, leading to improved methane yields compared to conventional systems. A semi-quantitative FMEA within a comprehensive risk framework suggests that the nano-CaO_2_/BC intervention could markedly reduce the RPN for critical failure modes such as acidogenic imbalance, ammonia toxicity, and H_2_S inhibition, potentially transforming TAD into a more stable process. For acidification, the severity remains at 9, but the drastic reduction in occurrence and improved detection lower the RPN by 85.7%. Furthermore, the technology enables closed-loop resource recovery: the resulting digestates, enriched with recalcitrant carbon and essential nutrients, hold promise as a high-value soil amendment that enhances fertility and promotes carbon sequestration. A technical break-even analysis indicates that positive net benefits are achievable through increased biogas recovery and avoided environmental penalties, provided that additive costs are lowered and co-benefits are fully monetized. Crucially, all quantitative claims in this review are theoretical projections derived from analogous systems; empirical validation through the proposed three-phase experimental framework is essential to confirm the synergistic mechanisms and performance projections. Ultimately, nano-CaO_2_/BC-enhanced TAD operationalizes a circular bioeconomy model, harmonizing renewable biogas generation with sustainable agriculture and establishing a transferable blueprint for managing high-strength organic waste streams.

## Figures and Tables

**Table 1:** Characteristics of tofu wastewater from different soybeans [2].

Substrate	TSS (mg/L)	COD (mg/L)	BOD (mg/L)	NH_3_-N (mg/L)
Red seed	392	4516.67	2375.63	8.79
Pati seed	218	4016.67	2254.74	8.61
Wonogiri seed	444	4583.33	3481.00	13.86
Green seed	344	4500.00	2976.06	8.73

(TSS: Total Suspended Solids; COD: Chemical Oxygen Demand; BOD: Biochemical Oxygen Demand; NH_3_-N: Ammonia Nitrogen).

**Table 2:** Wastewater characteristics from tofu processing facilities [4].

Processing Facility	pH	MLSS (mg/L)	BOD (mg/L)	COD (mg/L)	NH_3_-N (mg/L)	PO_4_-P (mg/L)	Turbidity (NTU)
Meurah Jaya	5.08	1600	4520.50	6400	64.00	2.56	921
Tahu Sumedang	5.50	3130	3810.20	5000	129.30	95.50	730
Tahu MKS	4.82	1050	4390.50	7300	39.90	1.57	902
Tahu Lampaseh Aceh	4.90	1177	3575.50	6500	36.10	1.81	387
Tahu Solo	4.85	1150	4415.50	8500	33.50	0.97	841

(MLSS: Mixed Liquor Suspended Solids; PO_4_-P: Orthophosphate; NTU: Nephelometric Turbidity Unit).

**Table 3:** Physicochemical properties of biochar from different feedstocks [18,40].

Feedstock	Temperature (°C)	Yield (%)	pH	Pore Volume (cm^3^/g)	Surface Area (m^2^/g)	C (%)	N (%)	O (%)	H (%)
Wheat straw	700	22.80	9.20	0.058	107.00	73.90	1.20	14.60	1.30
Soybean stover	300	37.03	7.27	—	5.61	68.81	1.88	24.99	4.29
Corn straw	500	29.30	10.40	0.012	6.00	58.00	2.30	21.50	2.70
Peanut shells	600	28.50	9.60	0.110	185.00	71.90	1.60	15.00	2.00
Soybean stover	700	21.59	11.32	0.19	420.30	81.98	1.30	15.45	1.27
Corn straw	400	35.50	10.20	0.008	4.00	56.10	2.40	22.00	4.30
Peanut shells	700	21.89	10.57	0.20	448.20	83.76	1.14	13.34	1.75
Wheat straw	500	27.60	8.30	0.090	111.00	70.30	1.40	17.70	2.90
Peanut shells	400	36.80	9.30	0.007	5.00	58.40	1.80	21.00	3.50
Corn straw	600	26.70	10.40	0.012	7.00	58.60	2.00	18.70	2.00
Peanut shells	300	36.91	7.76	—	3.14	68.27	1.91	25.89	3.85
Wheat straw	400	32.40	8.20	0.012	10.00	57.80	1.50	21.60	3.20

**Table 4:** Important reactions occurring during CaO_2_ catalytic biomass pyrolysis with thermodynamic data and risk-mitigation functions.

Reaction Name	Chemical Equation	ΔG at 700 °C (kJ/mol)	Risk-Mitigation Function
CaO_2_ decomposition	2CaO_2_ + Heat → 2CaO + O_2_	−296.8	Oxygen release for tar cracking and pore formation
Biomass volatiles + O_2_	C_x_HᵧO_2_ + O_2_ → CO + CO_2_ + H_2_O + hydrocarbons	−450 to −550	Tar reduction, syngas quality improvement
Primary water gas	C (from biochar) + H_2_O + Heat → CO + H_2_	+135.0	Hydrogen-rich syngas generation
Secondary water gas shift	CO + H_2_O ⇌ CO_2_ + H_2_	−28.6	H_2_/CO ratio adjustment
Steam Reforming of Tar	Tar (e.g., C_6_H_6_) + 6H_2_O → 6CO + 9H_2_	+356.2 (endothermic)	Tar elimination, enhanced biochar purity
Oxidative cracking	Tar + O_2_ → CO + CO_2_ + H_2_O	−550 (exothermic)	Complementary tar removal pathway
Carbonation	CaO + CO_2_ → CaCO_3_ + Heat	−130.4	CO_2_ sequestration, CaCO_3_ formation for sustained alkalinity

**Table 5:** Failure mode and effects analysis for conventional TAD of TWW.

Failure Mode	Severity (S)	Occurrence (O)	Detection (D)	RPN	Risk Classification
Acidification failure	9	8	7	504	High probability, high impact
Ammonia inhibition	8	6	8	384	Medium probability, high impact
H_2_S toxicity	7	5	9	315	Medium probability, high impact
VFA accumulation	8	7	6	336	High probability, medium impact
Pathogen survival	6	2	5	60	Low probability, medium impact
Heavy metal mobilization	5	3	7	105	Low probability, medium impact

**Table 6:** Projected failure mode and effects analysis for Nano-CaO_2_/BC-enhanced TAD of TWW.

Failure Mode	Severity (S)	Occurrence (O)	Detection (D)	RPN	Projected RPN Reduction (%)
Acidification failure	9 → 9	8 → 2	7 → 4	504 → 72	85.7
NH_3_ inhibition	8 → 5	6 → 2	8 → 5	384 → 50	87.0
H_2_S toxicity	7 → 4	5 → 2	9 → 4	315 → 32	89.8
VFA accumulation	8 → 5	7 → 3	6 → 4	336 → 60	82.1
Pathogen survival	6 → 4	2 → 1	5 → 4	60 → 16	73.3
Heavy metal mobilization	5 → 3	3 → 2	7 → 5	105 → 30	71.4

**Table 7:** Comprehensive and technical comparison of TAD enhancement strategies for high-strength organic waste.

Strategy	Key Mechanism(s)	Reported Methane Enhancement	Ammonia Nitrogen (NH_4_^+^-N) Control	Hydrogen Sulfide (H_2_S) Control	pH Buffering	Nutrient Recovery	References
Ca(OH)_2_ pretreatment	- Alkaline hydrolysis of lignocellulosic matrix - Partial solubilization of organic matter	15–25% (or up to 31% for food waste)	None	None	Limited to transient alkalinity consumption	None	[49,64]
Conventional alkaline (Ca(OH)_2_)	- Alkaline disruption of lignocellulosic biomass - Accelerated VFA conversion - Partial NH_4_^+^-N stripping via pH elevation to >10 (transient)	20–84% (range depending on substrate and duration)	Not quantified; possible stripping effect at high pH	Not reported	Consumption of alkalinity during VFA accumulation	Ca^2+^/PO_4_^3−^ precipitation as Ca_5_(PO_4_)_3_(OH)	[22,58]
Trace element supplementation	- Provision of essential enzyme cofactors (metallocofactors) - Enhances F_420_, MCR, hydrogenase activity - Critical for VFA metabolism (Co, Ni, Mo, Se, W)	30–40% (Mo, Se); 45–65% (mixed Co, Mo, Ni, Se, W) for low-background inocula	None directly (NH_3_ toxicity may be indirectly alleviated by improved metabolism)	None directly	Indirect via lower VFA accumulation	Not reported	[66]
Pristine biochar	- Physical adsorption of inhibitors (phenols, NH_4_^+^) - Conductive support matrix enabling DIET via electron tunneling - pH buffering via surface functional groups (-COOH, -OH)	18–37%	Moderate (via physical adsorption of NH_4_^+^; limited capacity ~10 mg/g)	Moderate (physical trapping; H_2_S oxidation by redox-active groups)	Mild (carboxyl/phenolic groups accept H^+^)	Low (surface groups bind K^+^, Ca^2+^, Mg^2+^)	[12,67]
Fe_3_O_4_-modified biochar	- Enhanced DIET due to Fe^2+^/Fe^3+^ redox cycling - Magnetic biochar facilitates conductivity without chemical or osmotic stress - Ferric iron (Fe^3+^) serves as an alternate electron acceptor	25–56% (or 62.6% under high-NH_4_^+^ stress for nano-Fe_3_O_4_)	Moderate (surface Fe^3+^ can bind NH_4_^+^)	No direct effect	No (Fe_3_O_4_ neutral); but mild effect if Fe^3+^ consumed	None reported	[47]
Mg/Fe-modified biochar	- Highly porous Mg-Fe oxide layer (lamellar double hydroxide, LDH) on biochar surface - Strong chemisorption of NH_4_^+^ via Mg^2+^ sites and interlayer exchange - High Fe^3+^/Fe^2+^ conductivity for DIET	116–229% (chicken manure at 2–5% additive)	High (87.83 mg NH_4_^+^-N/g) via LDH ion-exchange capacity	Low	No (neutral to slightly basic)	High (Mg^2+^ and PO_4_^3^- struvite precipitation potential)	[45]
KOH-activated biochar	- Alkaline activation creates ultra-high surface area, micropore volume, and graphitic degree - Boosted electron transfer (low resistance; high specific capacitance) - Promotes DIET between fermenters (Smithella) and methanogens (Methanosaeta)	52% increase in methane production rate [rate, not final yield] (Pennisetum giganteum)	Indirect: higher microbial activity improves NH_4_^+^ consumption	Not reported	No (process stability via reduced VFA lag)	Not assessed	[68]
FeCl_3_-impregnated biochar	- Fe^3+^ loading onto biochar surface enhances DIET via Fe^2+^/Fe^3+^ cycling - Promotes more diverse methanogenic pathways (methanol, dimethylamine, methylamine) - Enhances direct interspecies electron transfer	22.5% and 12.8% cumulative methane yield relative to control	Not quantified	Not quantified	No (Fe^3+^ slightly acidic)	No (Fe^3+^/Fe^2+^ redox)	[69]
Metal-engineered biochar	- Multi-metal (Fe, Ni, Co, Mn)-loaded via mechanochemistry: • Metal-carbon redox-active interfaces • Mesoporous structure enhances cell colonization - Spatial niche partitioning: BC phase drives DIET via Methanosarcina; sludge phase retains IHT (hydrogenotrophic pathway) - Fe-Ni cofactor activates key metalloenzymes (e.g., MCR)	cCH_4_ 224.7 NmL/g.VS Lag phase shortened from 22.3 days → 3.7 days	Not reported	Not reported	Not reported	Not reported	[70]
Nano zero-valent iron/biochar (nZVI/BC)	- nZVI undergoes corrosion (Fe^0^ → Fe^2+^ → Fe^3+^) generating reactive oxygen species + H_2_ (micro-aeration) - H_2_ promotes hydrogenotrophic methanogenesis - Fe^2+^/Fe^3+^ for direct DIET - Biochar support prevents nZVI agglomeration and toxicity	29.2–46.7% (vs. nZVI alone: 24–30%)	Not quantified	Very high: H_2_S concentration reduced from 8.32 mL to 0.22 mL (J) via precipitation of FeS (insoluble)	Not quantified	No (Fe^2+^/Fe^3+^ cycles)	[69]
MnO_2_-modified biochar	- MnO_2_ (Mn^4+^/Mn^2+^) redox capability enhances biochar capacitance → facilitates extracellular electron transfer - Promotes syntrophic fatty acid oxidation (Syntrophomonas) and methanogenesis (Methanosaetaceae)	24.3% (food waste); 12.7% (high NH_4_^+^-N, 2 g/L); 9.4% (high organic load, 30 g/L)	Moderate: 12.7% improvement under high NH_4_^+^ stress	Not quantified	Not directly (MnO_2_ neutral)	Not reported	[71]
Nano-CaO_2_/BC (projected)	- CaO_2_ → slow hydrolysis (H_2_O_2_) → oxidative stress and partial hydrolysis of recalcitrant polymers - OH^−^ released during CaO_2_ decomposition provides sustained pH buffering - Ca^2+^ precipitates excess PO_4_^3−^ → Ca_5_(PO_4_)_3_(OH) and possibly MgNH_4_PO_4_·6H_2_O - Biochar matrix facilitates adsorption, microbial colonization, and electron transfer	Estimated 30–50% (based on synergistic effects)	High (adsorption + oxidation + pH shift) not yet quantified	High (oxidation + precipitation of metal sulfides) projected	Sustained (slow hydrolysis of CaO_2_ over 2–4 weeks)	High-PO_4_^3−^ recovered as Ca_5_(PO_4_)_3_(OH) and possibly Mg-NH_4_-PO_4_ from additional Mg	Projected based on [13,14,17,19,20,54,60,62]; this review

## Data Availability

All data generated or analyzed during this study are included in this manuscript.
